# Addressing Racism in Medicine Through a Resident-Led Health Equity Retreat

**DOI:** 10.5811/westjem.2020.10.48697

**Published:** 2020-11-20

**Authors:** Anita N. Chary, Melanie F. Molina, Farah Z. Dadabhoy, Emily C. Manchanda

**Affiliations:** * Harvard Affiliated Emergency Medicine Residency, Boston, Massachusetts; † Boston Medical Center, Department of Emergency Medicine, Boston, Massachusetts

## Abstract

Racism impacts patient care and clinical training in emergency medicine (EM), but dedicated racism training is not required in graduate medical education. We designed an innovative health equity retreat to teach EM residents about forms of racism and skills for responding to racial inequities in clinical environments. The three-hour retreat occurred during the residency didactic conference to maximize resident participation. We prioritized facilitated reflection on residents’ own experiences of race and racism in medicine in order to emphasize these concepts’ relevance to all participants. We used workshop, small group, and panel formats to optimize interactivity and discussion. Post-retreat survey respondents indicated that the curriculum successfully promoted awareness of racism in the workplace. Participants also expressed interest in continued discussions about racism in medicine as well as desire for greater faculty and nursing participation in the curriculum. Residency programs should consider incorporating similar educational sessions in core didactic curricula.

## BACKGROUND

Racism refers to prejudice or discrimination directed against an individual or group on the basis of race.^1^ Patient care and healthcare providers’ experiences of the workplace are affected by racism.^2^ Resident physicians encounter racial discrimination directed toward patients and providers throughout clinical training.^3^–^5^ Discrimination may be amplified in the acute care environment of emergency medicine (EM). Implicit bias, or learned stereotypes that operate unconsciously and automatically, can be exacerbated in times of stress, when working with limited information, and in time-pressured situations—factors often present in emergency care.^6^

While some curricula exist to teach EM residents about social determinants of health and implicit bias,^7^–^9^ dedicated didactic teaching about racism in medicine is not required in EM residency curricula or in graduate medical education more broadly. We describe an innovative health equity retreat designed to teach residents about racism and its manifestations in medicine.

## OBJECTIVES

The educational objectives of the retreat were the following: 1) to raise awareness of race-based inequities in patient and resident experience; and 2) to build residents’ skills in recognizing and addressing racial inequities and microaggressions. To maximize opportunities for resident participation, the retreat was held during mandatory residency didactic conference, during which junior residents were protected from clinical duties. The retreat aimed to encourage peer discussions about racism and the potential roles of clinicians, bystanders, and allies in promoting equitable patient care and an inclusive training environment.

## CURRICULAR DESIGN

Four senior residents leading the residency’s Social Emergency Medicine group designed the retreat after conducting a literature review of existing EM curricula on race^7^–^9^ and holding working group meetings. A deliberate decision was made to base the retreat on the facilitated reflection of our own residents’ experiences of race, racism, and disparities in their specific clinical practice environments in order to emphasize these concepts’ relevance to all participants. Educators developed the curriculum around three major learning objectives: 1) understand definitions and examples of different forms of racism in emergency medicine; 2) understand the definitions and impacts of microaggressions; and 3) understand how provider bias impacts patient care. Retreat leaders assessed participant achievement of these objectives through a post-event survey.

Prior to the retreat, residents were invited to anonymously submit written accounts of how race and racism have affected them, their colleagues, and their patients in the emergency department (ED), through an online form. Eighteen of 58 residents submitted experiences, which were used as the basis for delivering the content of the retreat. At the time of the retreat, the racial composition of residents in the program was 63% White, 9% Black, 7% Latinx, and 21% Asian. The residency is based in an urban area of the Northeast United States, with residents reporting origins from the Northeast (42%), Southeast (2%), Southwest (8%), West (17%), Midwest (15%), Puerto Rico (2%), and foreign countries (10%).

The health equity retreat was comprised of three one-hour sessions held with 56 participants (40 of 58 residents, 16 faculty members). Eighteen residents were not present at the retreat, due in part to vacation periods and senior residents’ clinical obligations. The first session was an interactive presentation about forms of racism, led by a resident with a professional background in anthropology and race theory. Audience members, selected at random, were given envelopes containing quotations drawn from experiences submitted by their co-residents. The lecturer then called on these audience members to stand up and read the quotes aloud. The lecturer subsequently unpacked each quote to explain how the quote embodied key concepts about race. Quotes and paraphrased examples are included in Table 1, with permission from submitting residents.

The second session was a workshop devoted to microaggressions, defined as brief, commonplace words or actions (intentional or unintentional) that communicate hostility to or insult members of marginalized groups.^10^ Following a 15-minute didactic presentation, pairs of senior residents led small group discussions with residents and faculty members. Individual residents within each pair held different racial and gender identities. Discussion included the distinction between the intent and the impact of microaggressions. Specific cases were reviewed in which individuals’ intentions were benign, e.g., telling a resident, “You’re so well-spoken!” or confusing one resident of color for another. Small groups discussed the implications of these statements—in these examples, that people of a certain race are not expected to be well-spoken, or that residents of color are seen as interchangeable—and explored their negative impact on clinicians, particularly when occurring as a repeated experience. Strategies for responding to microaggressions were also reviewed, which have been published elsewhere.^5^

The third session reviewed the impact of patient race on the management of agitated patients in the ED. Example cases compared and contrasted management of White and Black patients with similar levels of agitation, including differential application of restraints and involvement of hospital security. A panel of EM attendings, social workers, and psychiatrists were invited to provide commentary. Resident facilitators then reviewed an algorithm they created to promote unbiased de-escalation of agitated patients in the ED (Appendix A). In cases where an immediate threat to physical safety is absent, the algorithm encourages clinicians to perform a brief internal assessment about whether race could be contributing to perceptions of threat, and provides specific strategies to facilitate verbal de-escalation.

## IMPACT/EFFECTIVENESS

An anonymous survey evaluating the retreat was sent to the 56 participants. Closed-ended questions assessed perceived utility of the sessions in teaching key concepts about race, microaggressions, and bias, using a five-point Likert scale (1=not at all useful, 2=slightly useful, 3=moderately useful, 4=very useful, 5=extremely useful), as well as desired topics for future instruction. Open-ended feedback about reactions to the sessions was solicited as well. Of 56 participants, 29 took the survey for a response rate of 52%. Respondents included 22 residents and 7 faculty. All participants (100%) reported improved understanding of diversity within the workplace. The majority (94%) found the sessions very or extremely useful. Survey respondents requested further training in related topics, including addressing discrimination from patients (83%), best practices in hallway care (83%), and implicit bias (59%). Two major themes were identified. The first was a need for continued discussion. The second was a desire for involvement of other key stakeholders, including faculty and nursing.

The health equity retreat was effective at promoting residents’ and faculty members’ awareness of racism in the workplace. Sharing of residents’ experiences followed by facilitated reflection was well received in each session. The retreat format aligns with the educational theory of experiential learning, in which concrete experience forms the basis for reflection, which in turn motivates changes in one’s approach to future actions or situations.^11^

Limitations in assessment of the retreat included low survey response rate and the fact that outcomes measured were self-reported, rather than indicative of attainment of learning objectives. However, an important lesson learned from the retreat was that this type of event created an opening for conversations about race and racism in EM, which resident and faculty participants have repeatedly reflected to the retreat organizers outside of the survey since the event.

The inaugural retreat led into a longitudinal health equity curriculum occurring over the rest of the academic year. This curriculum has included a series of lectures, panels, and journal clubs about health disparities and equity in the local practice environment, the evaluation of which is ongoing. Notably, not all residencies have affiliated members with backgrounds in race theory, and we suggest collaborating with educators with topical expertise as we are doing in the continued curriculum. Additionally, this one-time survey did not gauge long-term effectiveness of the workshop. For the next residency health equity retreat, we plan to perform surveys before, and at one and three months after the event, to improve understanding of the event’s longer term effects on self-reported behaviors. Future research is needed to understand how this retreat and similar educational efforts impact individuals’ behaviors toward colleagues and patients.

## Supplementary Information



## Figures and Tables

**Table 1:** Key concepts of racism illustrated through resident experiences.

Concept	Definition	Example
Interpersonal racism	Expression of racism between individuals, such as harassment, racial slurs, racial jokes, or singling someone out on basis of race	A patient tells the resident, “I don’t want a Black doctor.”
Institutional racism	Discriminatory treatment, unfair policies and practices, and inequitable opportunities within organizations or institutions based on race	Patients of color with congestive heart failure are disproportionately admitted to medicine, rather than cardiology.^13^
Internalized racism	Viewing oneself or one’s group through dominant prejudices about the inferiority of people of color	A resident reported preference to introduce self by first name, as “I was afraid that [my patients] would find out I am Hispanic and feel that they were being shortchanged, or even refuse my care.”
Stereotype	A standardized mental picture held in common about members of a group, representing an oversimplified opinion or judgment, without regard to individual difference	A clinician comments that a Latinx patient presents with ‘total body dolor’ and is in ‘status hispanicus.’
Implicit Bias	Learned stereotypes and prejudices that operate automatically, and unconsciously, when interacting with others, regardless of one’s intentions	An attending physician comments that a Black patient with sickle cell disease is “drug-seeking” and discourages the resident from ordering opioids.
Microaggression	Brief, commonplace words or actions, intentional or unintentional, that communicate hostility to or insult members of marginalized groups	“50% of my patient encounters include the patient/patient’s family questioning where I went to college and medical school.”
Privilege	Advantages and immunities enjoyed by one usually powerful group or class, to the disadvantage of others	“Every time I walk into the room with a female attending and the patient only makes eye contact with me [a white male resident].”

Definitions are adapted from the Boston Public Health Commission/Core Workshop.^12^
