# Supplementary figures and images for: Addressing Racism in Medicine Through a Resident-Led Health Equity Retreat
Source: West J Emerg Med. 2020 Nov 20;22(1):41–4. doi: 10.5811/westjem.2020.10.48697 (PMC7806337; doi:10.5811/westjem.2020.10.48697)

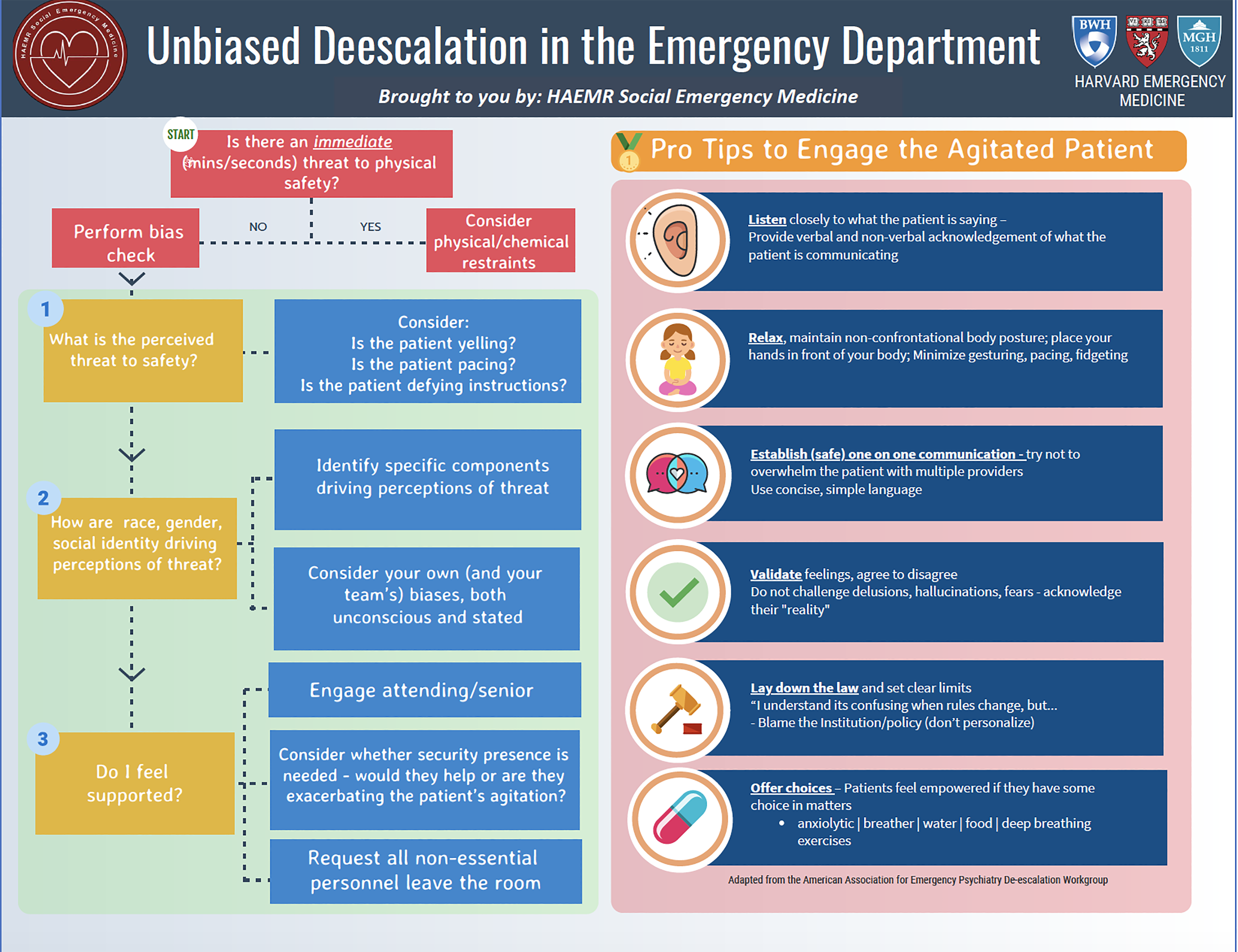

Supplement: Supplementary file 1 [file wjem-22-41-s001.png]
